# To Test or Not to Test: A Case Report on Hereditary Hemorrhagic Telangiectasia

**DOI:** 10.7759/cureus.55118

**Published:** 2024-02-28

**Authors:** Bhavya Chadalavada, Ritesh Baddam

**Affiliations:** 1 Internal Medicine, Gandhi Medical College, Hyderabad, IND

**Keywords:** autosomal dominant disorder, raynaud’s phenomenon, severe epistaxis, mixed connective tissue disease, hereditary hemorrhagic telangiectasia (hht)

## Abstract

A 50-year-old female patient presenting with joint pains, Raynaud’s phenomenon, epistaxis, and telangiectasias was posed with a diagnostic conundrum, i.e., whether to accept the diagnosis of mixed connective tissue disease (MCTD), for which she fulfilled all the criteria, or test for another probable disease, namely hereditary hemorrhagic telangiectasia (HHT), even though only some clinical features were present and all diagnostic criteria were not satisfied. Taking the patient’s onset of epistaxis as an important clue, the patient was counseled for genetic testing for HHT, which was positive. Treatment for both MCTD and HHT is underway, and appropriate surveillance is planned for the patient.

## Introduction

Hereditary hemorrhagic telangiectasia (HHT) is an autosomal dominant vascular disorder associated with mucocutaneous telangiectasias, epistaxis, arteriovenous malformations (AVMs) in the pulmonary, hepatic, and cerebral circulations, gastrointestinal bleeding, and iron deficiency anemia. Bleeding in HHT is due to malformed ectatic blood vessels, which results in increased fragility. Hereditary hemorrhagic telangiectasia is diagnosed using the Curaçao clinical criteria, which include family history, recurrent epistaxis, telangiectasia, and visceral AVMs. On the other hand, mixed connective tissue disease (MCTD) is a multisystem autoimmune disorder with overlapping features of at least two or more connective tissue diseases (CTD), including systemic sclerosis (SSc), systemic lupus erythematosus (SLE), polymyositis (PM), rheumatoid arthritis (RA), and dermatomyositis (DM). Here, we report the case of a 50-year-old woman presenting with epistaxis, telangiectasias, and clinical features suggestive of autoimmune disease.

## Case presentation

A 50-year-old female patient presented with complaints of recurrent epistaxis from six to seven years of age. It was initially mild but increased in severity and frequency over the past five years. She was found to have severe anemia and required several blood transfusions over the past five years. Furthermore, she had undergone nasal cauterization twice with no significant change in symptoms. Aside from this, she has no other bleeding manifestations. She complained of multiple red spots all over her body and inside her mouth as well.

The patient has a history of multiple joint pains, especially of the small joints, not associated with any swelling for the last four years. She noticed a change in the color of her fingers after exposure to cold water and in the winter season. She ignored these complaints as they weren’t causing her much distress as compared to the epistaxis. She has no history of photosensitivity, malar rash, or abortions. There is no history of cough, shortness of breath, dysphagia, or similar complaints in any other family members.

**Figure 1:**
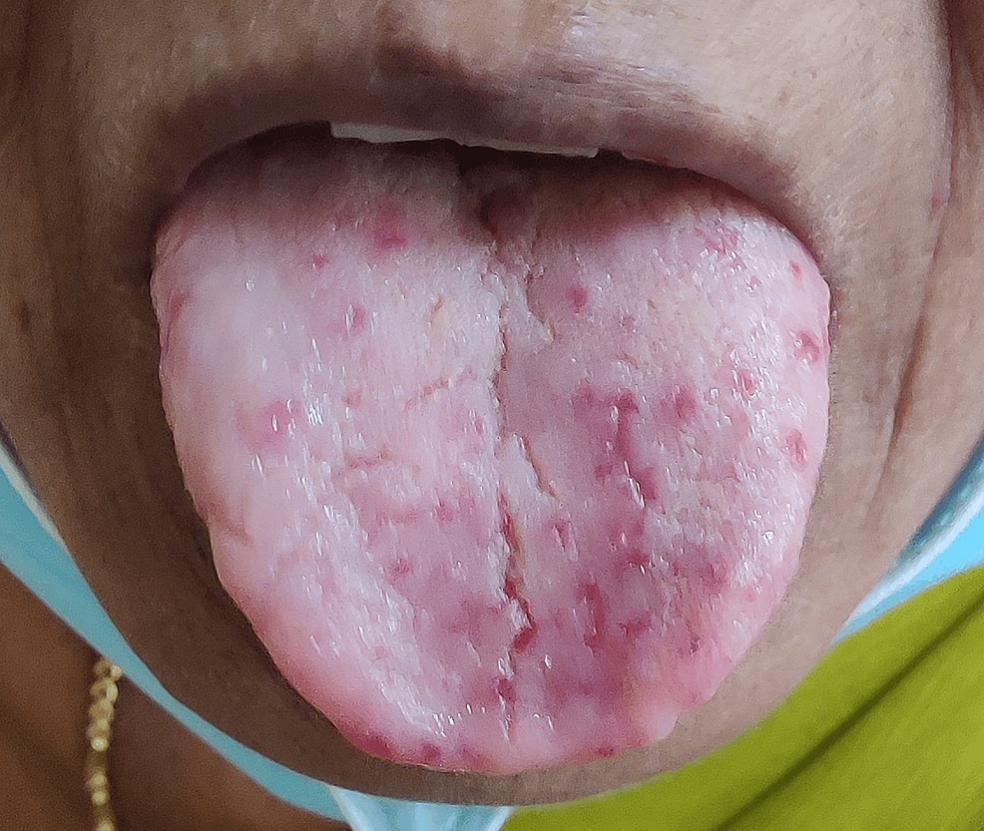
The 50-year-old patient with telangiectasias on the tongue

**Figure 2:**
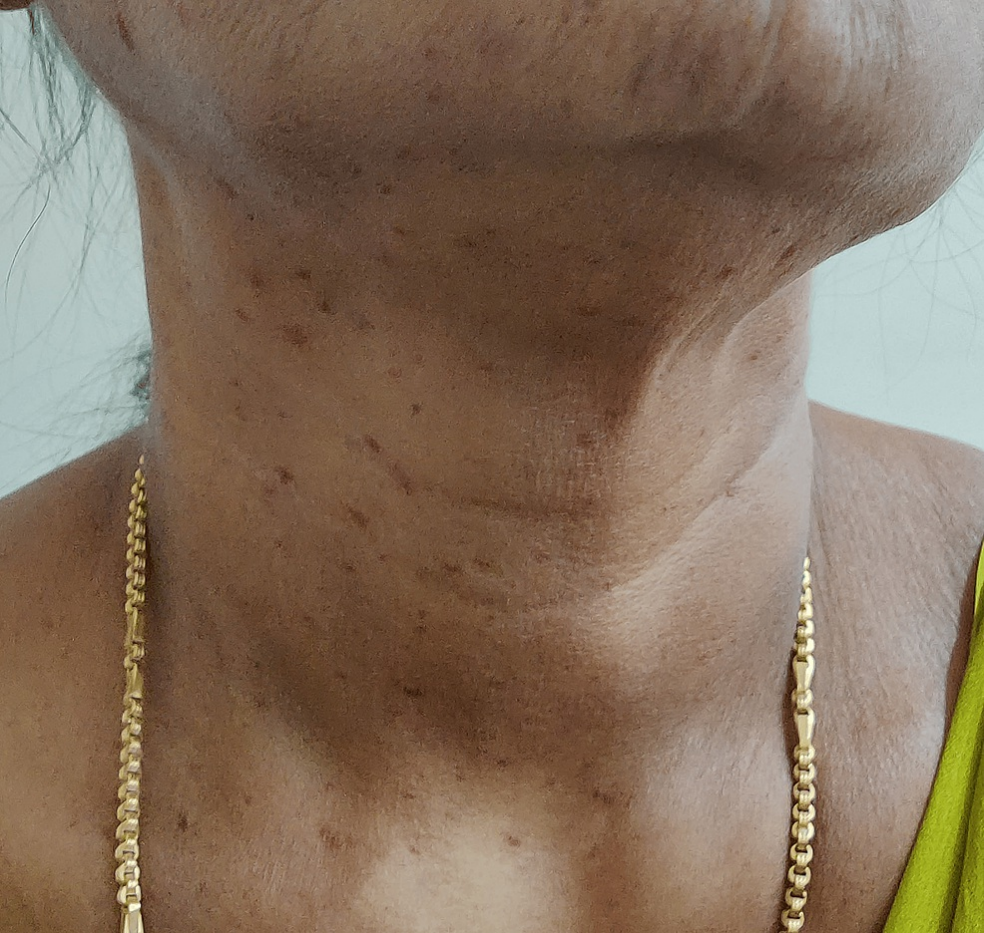
Multiple telangiectasias on the neck and chest

**Figure 3:**
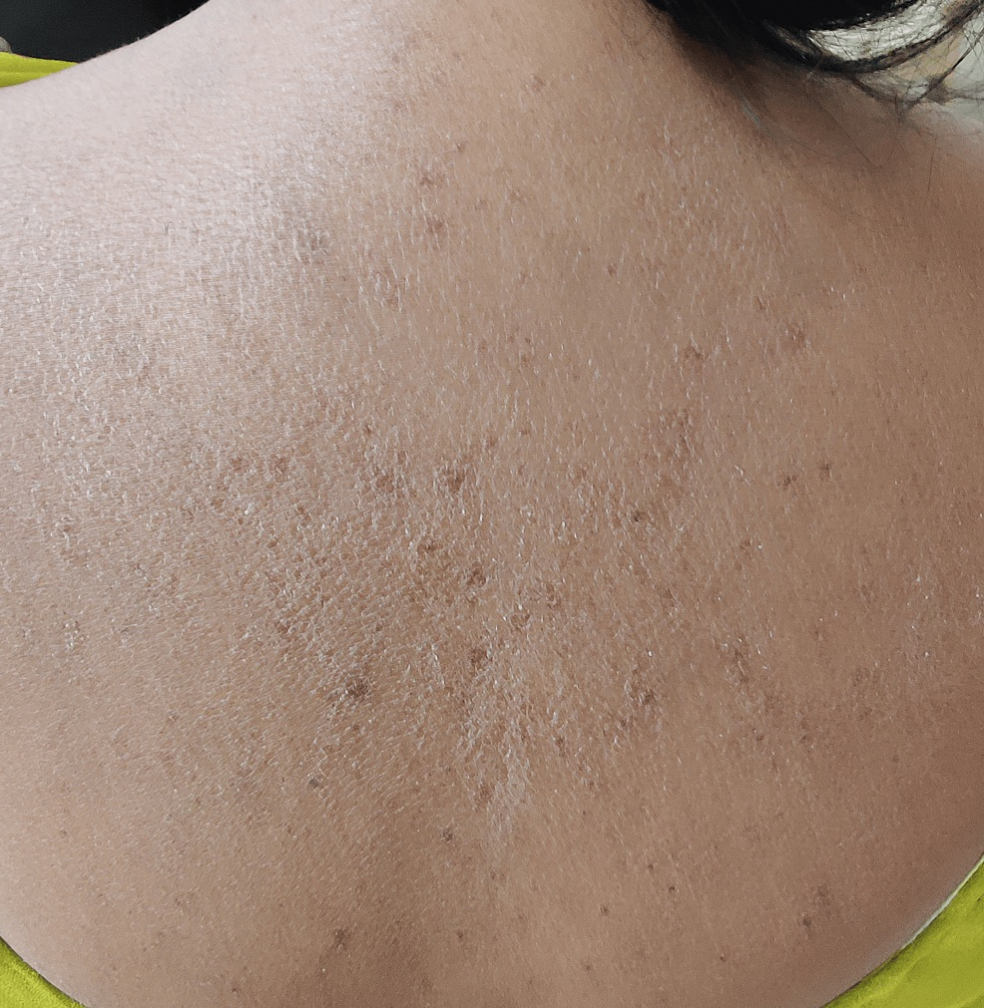
Multiple telangiectasias on the patient's back

On general examination, the patient was moderately built and nourished; her vitals were stable, and pallor was present. Small red macular lesions, around 1 mm in diameter, blanching on pressure suggestive of telangiectasias, were present over the face, tongue, neck, back, and palms (Figures 1-3). Similar red lesions were seen inside the oral and nasal cavities. There was mild tenderness over the small joints of her hands, with no obvious swelling or deformity. Raynaud’s phenomenon was induced by briefly immersing fingers in cold water. On systemic examination, no murmurs were heard, and the lungs were clear with no added sounds. Abdominal examination revealed no organomegaly. The neurological exam was normal.

On investigating the patient, hemoglobin was 7.5 g/dL, microcytic, hypochromic picture on peripheral smear, total WBC count was 5,600 cells/mm3, and platelet count was 225000/μL. Liver function and renal function tests were normal. The coagulation profile was normal. The stool was negative for occult blood. The antinuclear antibody (ANA) screening by immunofluorescence was a 4+, positive, speckled pattern; the ANA profile was strongly positive for anti-U1 ribonucleoprotein (RNP) antibodies. The chest X-ray, 2D echo, ultrasound abdomen, and upper GI endoscopy were normal, and a colonoscopy is pending. A high-resolution CT chest was done to look for interstitial lung disease (ILD), which was normal. An MRI of the brain was done to look for cerebral AVMs and was revealed to be normal. The creatine phosphokinase (CPK) level was borderline elevated at 358 IU/L. Results of spirometry, rheumatoid factor (RF) IgM, and anti-cyclic citrullinated peptide (CCP) are pending. Ultrasound of the bilateral hands showed synovitis of the proximal interphalangeal joints and metacarpophalangeal joints of both hands. Table 1 is a summary of the important lab investigations conducted on the patient. 

**Table 1:** Laboratory investigations ANA: Antinuclear antibody, CPK: Creatine phosphokinase, RNP: Ribonucleoprotein

Investigations	Results
Hb	7.5g/dL
Platelet count	225000μL
Total leukocyte count	5600/mm3
Total bilirubin	1.1mg/dL
Serum creatinine	0.9mg/dL
ANA	4+ speckled
ANA profile	Anti U1RNP +++
CPK	358 IU/L

A rheumatology opinion was taken regarding the autoimmune features in this patient, and a diagnosis of MCTD was made based on the recent 2019 criteria in Japan. Even though there was no family history and an absence of visceral AVMs on the scan, we suspected HHT and counseled the patient for genetic testing. The patient agreed to undergo a genetic evaluation, which revealed a heterozygous pathogenic variant in the ACVRL1 gene, thus confirming the diagnosis.

## Discussion

Mixed connective tissue disease has no unique clinical features, and there is a significant variation in clinical manifestations among individuals [1]. In the absence of family history and visceral AVMs, the diagnosis of HHT could have been missed. Even though our patient had joint pains, Raynaud’s phenomenon, and positive immunology criteria for MCTD, which is well known to be associated with telangiectasias, the patient’s onset of epistaxis at six to seven years of age prompted further investigation. There are some case reports of the association of HHT with autoimmune diseases like scleroderma and Hashimoto thyroiditis, although the cause of this association is unclear. 

Genetic testing is recommended to establish the diagnosis in patients who do not satisfy the Curaçao criteria, as described in Table 2 below, or in those who are asymptomatic or have minimal symptoms. Genetic testing can be done to identify the causal mutation in an individual who has been clinically confirmed to have HHT, as well as to test additional members of the patient's family who have a known causal mutation before symptoms manifest. If clinical features are not conclusive, a heterozygous pathogenic variant in ACVRL1, ENG, or SMAD4 can confirm the diagnosis through genetic testing [2].

**Table 2:** Curaçao criteria for the clinical diagnosis of HHT The diagnosis for HHT is definite if ≥3 criteria are present or if a pathogenic variant is identified in a known HHT gene; suspected if two of the criteria are present; and unlikely if <2 criteria are present. HHT: Hereditary hemorrhagic telangiectasia, AVM: Arteriovenous malformation

Criteria	Characteristics
Telangiectasia	Multiple characteristic sites: Lips, oral cavity, fingers, and nose
Epistaxis	Recurrent spontaneous nosebleeds
Visceral involvement	GI telangiectasia
	Pulmonary AVM
	Hepatic AVM
	Cerebral VM
	Spinal AVM
Family history	First-degree relative with known HHT: Parent, sibling, or child

All patients who have been diagnosed with or are suspected of having HHT should undergo screening. This includes yearly checks for signs and symptoms of the disease, evaluation for pulmonary AVMs every five years using transthoracic contrast echocardiography (TCE) in adults and chest radiographs with pulse oximetry or TCE in children, annual upper GI endoscopy, colonoscopy every three years beginning at age 15, and annually if colonic polyps are found in those with SMAD4-positive HHT [3].

Humidification, topical moisturizing therapy, hemostatics, antifibrinolytics, ablation therapy, systemic antiangiogenic drugs, septodermoplasty, and nasal closure may be used to treat nosebleeds when appropriate. Blood transfusions and iron replacement treatments are used to treat GI bleeding and anemia. Occlusion is usually necessary for pulmonary AVMs with feeding vessels that are 1 mm to 2 mm in diameter or larger to prevent strokes. Hepatic AVMs have few treatment options because embolization frequently causes further liver-related complications. Monitoring is necessary for encephalopathy, pulmonary hypertension, portal hypertension, and high-output heart failure in patients with hepatic AVMs. Currently, available treatment options include systemic medications such as bevacizumab or orthotopic liver transplantation. Surgery, embolotherapy, and stereotactic radiosurgery are used to treat cerebral AVMs.

The diagnosis of MCTD is made per the 2019 criteria issued by the Japan Research Committee of the Ministry of Health, Labor, and Welfare for systemic autoimmune diseases (Table 3) [4]. Mixed connective tissue disease is diagnosed when a patient satisfies all of the criteria listed, i.e., at least one common manifestation, an immunological manifestation, and at least one characteristic organ involvement. Or, when a patient satisfies at least one common manifestation, an immunological manifestation, and at least one feature from two or more of the overlapping manifestations listed under A, B, and C in Table 3 below [4].

**Table 3:** The 2019 diagnostic criteria for MCTD MCTD: Mixed connective tissue disease, RNP: Ribonucleoprotein

Diagnostic criteria	Characteristics
I. Common manifestations	1. Raynaud’s phenomenon
	2. Puffy fingers and/or swollen hands
II. Immunological manifestation	Positivity for anti-U1 RNP antibody
III. Characteristic organ involvement	1. Pulmonary arterial hypertension
	2. Aseptic meningitis
	3. Trigeminal neuropathy
IV. Overlapping manifestations	
A. Systemic lupus erythematosus-like manifestations	1. Polyarthritis
	2. Lymphadenopathy
	3. Malar rash
	4. Pericarditis or pleuritis
	5. Leukopenia (4,000/μL or less) or thrombocytopenia (100,000/μL or less)
B. Systemic sclerosis-like manifestations	1. Sclerodactyly
	2. Interstitial lung disease
	3. Esophageal dysmotility or dilatation
C. Polymyositis/dermatomyositis-like manifestations	1. Muscle weakness
	2. Elevated levels of myogenic enzymes
	3. Myogenic abnormalities on electromyogram

Our patient met these criteria and was diagnosed with MCTD along with HHT. The treatment of MCTD is similar to that of SLE, SSc, PM, or DM in many ways. She was initially started on a low-dose steroid for arthritis and nifedipine 10 mg, which was further escalated to 30 mg extended-release nifedipine for Raynaud's, following which she improved symptomatically. Since the epistaxis persisted, she was referred to an ENT surgeon for consideration of ablative procedures.

There are case reports of telangiectasias as the sole presentation of MCTD [5,6], and this case could have easily been mistaken for the same. The treating physicians need to have a strong clinical suspicion for HHT and avoid bias. This would enable the correct diagnosis of the patient and the screening of asymptomatic family members, especially in the absence of a family history.

## Conclusions

In the literature, telangiectasias have been reported as the singular presentation of MCTD. However, this was not the case with our patient. The MCTD presents with multi-system involvement, and prominent cutaneous features can often be confused with other diseases. Hence, when in doubt, other diagnoses must be considered, like in our case. An early diagnosis of HHT results in the timely detection of systemic AVMs, which allows for prophylactic treatment, prompt screening, and the prevention of complications. It is imperative for treating physicians to have a strong clinical suspicion for HHT. This would help avoid bias and ensure the correct diagnosis of the patient. Furthermore, especially in the absence of a family history, screening for HHT must be recommended for asymptomatic family members.
